# Fellowships, Grants, & Awards

**Published:** 2007-01

## CAM at Minority or Health Disparities Research Centers (R21)

The purposes of this initiative are to: 1) stimulate high-quality, preliminary studies of complementary and alternative medicine (CAM) at institutions committed to minority health research or health disparities research, as evidenced by having received a grant from National Institutes of health (NIH) or the Agency for Healthcare Research and Quality (AHRQ) for a research center on minority health or health disparities relating to racial and ethnic minority populations; 2) increase the knowledge base regarding CAM and health disparities; 3) attract investigators experienced in minority health and health disparities research to the field of CAM; and 4) provide a stable scientific environment where CAM practitioners can participate actively in rigorous research.

The purpose of this solicitation is to stimulate research that will enhance the understanding of the mechanisms of CAM interventions and increase the knowledge base regarding the potential role of CAM practices, including traditional indigenous medicine practices, either in reducing and eliminating health disparities, or regarding minority health. This program announcement will support exploratory/developmental (R21) research projects on CAM interventions at institutions that have received NIH or AHRQ awards for research centers on minority health or health disparities. The infrastructure and resources of these centers may be used to support basic, preclinical, clinical, translational, and health services research projects addressing issues relating to CAM and minority health or health disparities. For the purpose of this program announcement, an eligible institution is defined as one that has received funding from NIH or AHRQ for a single organizational entity incorporating multiple research projects and associated project cores. The list of program announcements from which eligible centers are funded may be found at the website (http://nccam.nih.gov/announcements/disparitiesPAR.htm).

The National Center for Complementary and Alternative Medicine (NCCAM) defines CAM practices as those that are “unproven by science and not presently considered an integral part of conventional medicine” (http://nccam.nih.gov/about/plans/2005/index.htm). NCCAM groups the wide range of CAM modalities into four domains: 1) mind–body medicine; 2) biologically based practices; 3) manipulative and body-based practices; and 4) energy medicine. In addition, NCCAM studies whole medical systems, such as Ayurvedic medicine, traditional Chinese medicine, traditional indigenous medicine, and homeopathy. Mind–body interventions use a variety of techniques designed to enhance the mind's capacity to affect body functions and symptoms.

Mind–body techniques that are considered CAM include prayer, mental healing, and therapies that use creative outlets such as art, music, or dance, and meditation for selected purposes. Other techniques that were considered CAM in the past have become mainstream, such as patient support groups and cognitive-behavioral therapy.

Biologically based practices for prevention and therapy use foods and their components, diets, and dietary supplements such as vitamins, herbs, and other natural products.

Manipulation and body-based methods in CAM are based on manipulation and/or movement of one or more parts of the body. Examples include chiropractic or osteopathic manipulation, and massage therapy.

Energy therapies are of two types: 1) biofield therapies (e.g., qi gong, Reiki, and therapeutic touch), which are intended to affect energy fields that surround and penetrate the human body, and 2) bioelectromagnetic-based therapies that involve the unconventional use of electromagnetic fields such as pulsed fields, magnetic fields, or alternating current or direct current fields for selected purposes.

Although numerous surveys document use of CAM by the general population, less information is available on CAM use by racial and ethnic minority populations. NCCAM previously published the initiative “Secondary Analysis of Data on CAM Use in Minority Populations” to stimulate research on this subject. Some practices now considered to be CAM have long histories of use as traditional practices in populations and cultures outside the mainstream. America's increasing cultural diversity and increasing minority populations necessitate a better understanding of their views on health, illness, and health-care. Because of the integral role of traditional indigenous/CAM practices in these cultures, a better understanding of these factors can provide insight into health behaviors and beliefs of the populations, generating information that may inform the delivery of conventional health care and lead to reduced health disparities.

At the same time, statistics document significant disparities for minority populations in health outcomes, such as quality of life, as well as mortality, processes, quality, and appropriateness of care, and the prevalence of certain conditions or diseases. DHHS has targeted health disparities in six conditions—HIV/AIDS, cancer, cardiovascular diseases, diabetes, adult and childhood immunizations, and infant mortality—for elimination by the year 2010, in response to which NIH has funded several research centers to address these and other health disparities. The centers funded by NIH for research on minority health and health disparities research, as well as the AHRQ centers for research on health disparities, all can contribute to the objectives of this program announcement.

While many CAM therapies are in extensive use by the public, few have been thoroughly tested for safety and/or efficacy. For this reason, the potential role of traditional or CAM practices in the elimination of health disparities remains to be defined. As part of its contribution to this effort, NCCAM seeks to fund research to determine the safety and efficacy of CAM interventions regarding minority health and the possible roles for CAM in contributing to the elimination of racial and ethnic health disparities. Research illuminating mechanisms of action for relevant CAM interventions as well as outcomes of CAM use for health disparities conditions or diseases also are of interest. NCCAM intends to build on research investments addressing health disparities or minority health by soliciting applications from those centers funded by NIH or AHRQ for research on minority health or health disparities. By stimulating high- quality, exploratory/developmental studies of CAM at institutions with infrastructure and research activities focusing on minority health or health disparities, NCCAM seeks to generate new knowledge regarding CAM as it relates to racial and ethnic health disparities.

Centers for research on minority health or health disparities span a broad research spectrum including basic, preclinical, clinical, translational, and health services research relating to minority health and health disparities. To increase the understanding of all aspects of traditional and CAM practices as they relate to health disparities, this program announcement invites institutions with NIH- or AHRQ-funded minority health centers or health disparities centers to submit applications on research topics relating to CAM and minority health or health disparities. Because NCCAM previously announced the initiative, Secondary Analysis of Data on CAM Use in Minority Populations (PAR-03-102), applications proposing epidemiologic studies of CAM use or secondary analyses of data on CAM use will not be considered responsive to this initiative.

The objective of this Funding Opportunity Announcement (FOA) is to encourage the submission of high quality exploratory/developmental studies investigating the spectrum of CAM and traditional indigenous medicine interventions as they apply to racial and ethnic minority populations or health disparities. Applications may propose research projects for basic, preclinical, clinical, translational, or health services research studies of CAM or traditional indigenous medicine interventions as they relate to minority health or health disparities. Those applications seeking funding for clinical research grants may propose projects in any CAM domain in order to provide preliminary data that can be used as a foundation for larger clinical studies and illuminate a possible role for traditional indigenous or CAM practices in reducing and ultimately eliminating health disparities in cancer, diabetes, cardiovascular diseases (including stroke), HIV/AIDS, or other minority health or health disparity condition. This FOA seeks to stimulate research that will increase the understanding of CAM in relation to minority health conditions or identified health disparities and identify possible roles for traditional or CAM practices in reducing and ultimately eliminating health disparities. Clinical studies proposing projects involving procedure-based CAM or traditional indigenous medicine practices are required to include a knowledgeable practitioner as part of the research team.

The following list of research topics provides examples of the types of research projects being sought by this initiative. This list is illustrative, is not exhaustive, is not intended to be exclusive, and is not in priority order. In addition, NCCAM has identified research areas of special interest as well as areas that are subject to a short “pause” in new research funding. Please see the NCCAM website (http://nccam.nih.gov/research/priorities/index.htm#5) for further information: 1) Mechanistic studies on the spectrum of CAM modalities that might be useful for health disparity diseases or conditions: biologically-based treatments, herbs, bioactive food components, nutritional supplements, natural products, homeopathy, manual therapies (such as spinal manipulation and mobilization as performed in chiropractic or osteopathic practices), bioenergy modalities such as qi gong, Reiki, distant healing, acupuncture, and others; 2) Mechanisms of action of complex botanicals focusing on the cellular, molecular, endocrine, and metabolic changes induced *in vitro*, in animal models, and in human subjects treated with these botanicals; 3) Interactions between CAM and conventional therapeutic modalities, including but not limited to those of complex botanicals with pharmaceutical drugs; 4) Projects that study the effects of CAM interventions (including botanical substances or other natural products) alone or in combination with conventional therapy on the progression of disease or clinical outcomes; 5) Studies on potential roles for CAM interventions (e.g., homeopathic remedies, various energy modalities, meditation, mind–body practices, etc.) in patients to slow the progression of disease, decrease symptoms, ameliorate medication side effects, or improve quality of life; 6) Projects to characterize and investigate the safety and efficacy of interventions from traditional medicine systems (e.g., traditional indigenous medicine, traditional Chinese medicine, Ayurveda, etc.) used to treat health disparity conditions/diseases; 7) Feasibility studies to test and optimize parameters such as accrual rates, acceptance of randomization, compliance, delivery of the intervention, appropriate inclusion/exclusion criteria, and optimization of the overall protocol design for planned clinical trials; 8) Studies to develop and validate testing of biological and behavioral outcome measures in humans for use in CAM clinical research. These could include development and/or validation of both the measurement procedure itself and delivery to subjects. Examples include optimization of imaging methods and enzymatic assays, and development and validation of behavioral assessment tools to be used in clinical trials; 9) Studies to determine if effect sizes described in the literature or clinically significant effect sizes can be achieved; 10) Small clinical studies to determine safety, toxicity, pharmacokinetics, pharmacodynamics, and the optimal dosage of an intervention as a prelude to a larger efficacy trial; 11) Qualitative research, such as detailed case studies and patient and health care provider interviews, ethnographic or ethnobotanical studies, to describe diagnostic and treatment approaches; to explore patient and health care provider preferences, beliefs and attitudes; and to investigate the relevance of those approaches to future clinical studies; 12) Studies of the effects of CAM interventions on patient adherence to conventional therapy; 13) Clinical studies of natural products, including functional foods, extracts, and their components, shown to have strong *in vitro* activity relating to a health disparity condition; 14) Studies of the impact of CAM interventions on objective and subjective measures of symptoms, well-being, or quality of life; 15) Studies to address issues regarding the impact of insurance availability and costs of CAM on CAM use and the impact of managed care on CAM use; 16) Outcomes studies that include measurement of nonclinical patient-oriented variables as well as clinical variables in assessing results, such as measures of the patient's health-related quality of life, patient satisfaction, personal preferences, and functional abilities: for example, measuring the effect of CAM use on the results of treatment; identifying health disparity conditions for which CAM use appears to influence outcomes either positively or unfavorably; determining whether CAM use affects patient satisfaction or makes a difference in the functional result of care, and whether CAM interventions can be linked causally to specific outcomes; 17) Studies that seek to clarify biomarkers of exposure to functional foods and their bioactive components, and determinants of biological response involved with minority health and health disparities.

This funding opportunity will use the NCCAM R21 (Exploratory/Developmental Project) award mechanisms.

As an applicant, you will be solely responsible for planning, directing, and executing the proposed project. Applicants are encouraged to direct inquiries regarding the appropriate mechanism to scientific/research staff listed in the Section VII.1. Scientific/Research Contacts.

The R21 awards are exploratory/developmental research grants for support of pilot and feasibility research designed to provide investigators with an opportunity to produce preliminary data in support of a larger project that may be submitted in the future as a research project (R01) grant application. Two NCCAM R21 funding mechanisms (http://nccam.nih.gov/research/instructions/r21/index.htm) are available: 1) Basic and Preclinical Research on Complementary and Alternative Medicine and 2) NCCAM Exploratory/Developmental Grant for Clinical Studies. Applicants are advised to check the website above for the most current NCCAM R21 announcements, and also to abide by the application guidance, allowable project period length, and direct cost limits.

This FOA uses just-in-time concepts. It also uses the modular budget formats (see the “Modular Applications and Awards” section of the NIH Grants Policy Statement. Specifically, if you are submitting an application with direct costs in each year of $250,000 or less (excluding consortium Facilities and Administrative [F&A] costs), use the PHS398 Modular Budget component provided in the SF424 (R&R) Application Package and SF424 (R&R) Application Guide (see specifically Section 5.4, “Modular Budget Component,” of the Application Guide).

Exploratory/developmental grant support is for new projects only; competing renewal (formerly “competing continuation”) applications will not be accepted. Up to two resubmissions (formerly “revisions/amendments") of a previously reviewed exploratory/developmental grant application may be submitted. See NOT-OD-03-041, May 7, 2003.

Applicants must download the SF424 (R&R) application forms and SF424 (R&R) Application Guide for this FOA through Grants.gov/Apply.

Note: Only the forms package directly attached to a specific FOA can be used. You will not be able to use any other SF424 (R&R) forms (e.g., sample forms, forms from another FOA), although some of the "Attachment" files may be useable for more than one FOA.

For further assistance, contact GrantsInfo: 301-435-0714, telecommunications for the hearing impaired: TTY 301-451-0088) or by e-mail: GrantsInfo@nih.gov.

The letter of intent receipt date for this PAR is 15 October 2007, with the application receipt date 14 November 2007. The complete version of this PA is available at http://grants.nih.gov/grants/guide/pa-files/PAR-06-372.html.

Contact: Morgan N. Jackson, Division of Extramural Research and Training, National Center for Complementary and Alternative Medicine, 6707 Democracy Boulevard, Suite 401, Bethesda, MD 20892-5475 USA (for courier service, use the Zip Code 20817 USA) 301-402-1278, e-mail: mj145m@nih.gov; Sharon Ross, Division of Cancer Prevention, National Cancer Institute, 6130 Executive Bouelvard, Room 3157, MSC 7328, Bethesda, MD 20892-7328 USA, 301-594-7547, fax: 301-480-3925, e-mail: rosssha@mail.nih.gov; Mireille Kanda, National Center on Minority Health and Health Disparities, National Institutes of Health, 6707 Democracy Boulevard, Suite 800, MSC-5465, Bethesda, Maryland 20892-5465 USA, 301-402-1366, fax: 301-480-4049, e-mail: kandam@mail.nih.gov.

Reference PAR-06-372.

## Research Project Grant (Parent R01)

The Research Project Grant (R01) is an award to support a discrete, specified, circumscribed project to be performed by named Project Directors/Principal Investigators (PDs/PIs) in areas representing the investigators’ specific interests and competencies, based on the mission of the NIH. The R01 is the original and historically the oldest grant mechanism used by the NIH to support health-related research and development.

The NIH awards R01 grants to institutions/organizations of all types. This mechanism allows the PDs/PIs to define the scientific focus or objective of the research based on particular areas of interest and competence. Although the PDs/PIs write the grant application and are responsible for conducting and supervising the research, the actual applicant is the research institution/organization.

Research grant applications are assigned to an NIH Institution or Center (IC) based on receipt and referral guidelines and many applications are assigned to multiple ICs with related research interests.

Each IC maintains a website with funding opportunities and areas of interest. Contact with an IC representative may help focus the research plan based on an understanding of the mission of the IC. For specific information about the mission of each NIH IC, see http://www.nih.gov/icd, which provides a brief summary of the research interests in each IC and access to individual IC home pages.

This Funding Opportunity Announcement (FOA) will use the Research Project Grant (R01) award mechanism.

The applicant will be solely responsible for planning, directing, and executing the proposed project.

This FOA uses just-in-time information concepts. It also uses the modular as well as the non-modular budget formats (see http://grants.nih.gov/grants/funding/modular/modular.htm).

Specifically, if you are a U.S. organization and are submitting an application with direct costs in each year of $250,000 or less (excluding consortium Facilities and Administrative [F&A] costs), use the PHS398 Modular Budget component provided in the SF424 (R&R) Application Package and SF424 (R&R) Application Guide (see specifically Section 5.4, “Modular Budget Component,” of the Application Guide).

United States applicants requesting more than $250,000 in annual direct costs and all foreign applicants must complete and submit detailed budget requests using the Research & Related Budget component found in the application package for this FOA. See NOT-OD-06-096, August 23, 2006.

Applicants must download the SF424 (R&R) application forms and the SF424 (R&R) Application Guide for this FOA through Grants.gov/Apply.

Note: Only the forms package directly attached to a specific FOA can be used. You will not be able to use any other SF424 (R&R) forms (e.g., sample forms, forms from another FOA), although some of the "Attachment" files may be useable for more than one FOA.

For further assistance, contact GrantsInfo: 301-435-0714, (telecommunications for the hearing impaired: TTY 301-451-0088) or by e-mail: GrantsInfo@nih.gov.

The application submission dates are available at http://grants.nih.gov/grants/funding/submission-schedule.htm.

The complete version of this PA is available at http://grants.nih.gov/grants/guide/pa-files/PA-07-070.html.

Contacts: The complete list of agency contacts is available at http://grants.nih.gov/grants/guide/pa-files/PA-07-070.html.

Reference: PA-07-070.

